# Heterologous Expression of the Pathogen-Specific LIC11711 Gene in the Saprophyte *L. biflexa* Increases Bacterial Binding to Laminin and Plasminogen

**DOI:** 10.3390/pathogens9080599

**Published:** 2020-07-22

**Authors:** Leandro Toshio Kochi, Luis Guilherme Virgílio Fernandes, Ana Lucia Tabet Oller Nascimento

**Affiliations:** 1 Laboratório de Desenvolvimento de Vacinas, Instituto Butantan, São Paulo 05503-900, Brazil; leandro.kochi@butantan.gov.br (L.T.K.); luis.fernandes@butantan.gov.br (L.G.V.F.); 2 Instituto de Ciências Biomédicas, Universidade de São Paulo, São Paulo 05508-900, Brazil

**Keywords:** *Leptospira*, leptospirosis, virulence

## Abstract

Leptospirosis is a febrile disease and the etiological agents are pathogenic bacteria of the genus *Leptospira*. The leptospiral virulence mechanisms are not fully understood and the application of genetic tools is still limited, despite advances in molecular biology techniques. The leptospiral recombinant protein LIC11711 has shown interaction with several host components, indicating a potential function in virulence. This study describes a system for heterologous expression of the *L. interrogans* gene *lic11711* using the saprophyte *L. biflexa* serovar Patoc as a surrogate, aiming to investigate its possible activity in bacterial virulence. Heterologous expression of LIC11711 was performed using the pMaOri vector under regulation of the *lipL32* promoter. The protein was found mainly on the leptospiral outer surface, confirming its location. The *lipL32* promoter enhanced the expression of LIC11711 in *L. biflexa* compared to the pathogenic strain, indicating that this strategy may be used to overexpress low-copy proteins. The presence of LIC11711 enhanced the capacity of *L. biflexa* to adhere to laminin (Lam) and plasminogen (Plg)/plasmin (Pla) in vitro, suggesting the involvement of this protein in bacterial pathogenesis. We show for the first time that the expression of LIC11711 protein of *L. interrogans* confers a virulence-associated phenotype on *L. biflexa*, pointing out possible mechanisms used by pathogenic leptospires.

## 1. Introduction

The genus *Leptospira* includes free-living saprophytic and pathogenic strains responsible for causing leptospirosis in mammals. Leptospirosis is distributed worldwide with approximately 1 million cases per year reported in humans [1]; outbreaks of this disease occur during the rainy season, with flooding, mainly in developing tropical countries [2].

Pathogenic leptospires enter the host through the skin or mucosal lesions and spreads rapidly through the bloodstream, reaching target organs. The early symptoms of the disease are similar to those of other febrile illnesses, so early diagnosis is difficult [3]. In humans, symptoms may evolve to a severe condition characterized by pulmonary hemorrhage with mortality rates over 50% in cases [4,5].

Outer membrane proteins (OMPs) of pathogenic *Leptospira* play important roles in different steps of pathogenesis, including adhesion to extracellular matrix components (ECM) and binding to immune system regulators and components of fibrinolytic and coagulation systems [6,7,8]. The majority of studies of these proteins are performed in vitro by heterologous expression in *Escherichia coli* and purification of the recombinant counterpart. Although many of these proteins are well characterized, we still have an incipient knowledge of their role in leptospiral pathogenesis, a limitation that impacts disease control and vaccine development.

Recently, the genetic tools to understand the role of leptospiral proteins have expanded, allowing heterologous expression of proteins of interest by *E. coli-Leptospira* shuttle vectors [9,10] and subsequent evaluation of gain of function phenotype. Moreover, gene knockout by random transposon [11,12] or homologous recombination [13], and more recently gene silencing using CRISPRi [14], have made it possible to study the loss of function phenotypes.

However, gene disruption or silencing in pathogenic strain have some drawbacks, due to functional redundancy displayed by these bacteria [15], making the observation of significant phenotype loss difficult. In addition, several target proteins have low copy numbers in *L. interrogans* [16], resulting in unmeasurable phenotypes after gene disruption, reducing the sensitivity of this strategy.

In this sense, the heterologous protein expression by a strong and constitutive promoter in the surrogate *L. biflexa* is evolving as an interesting approach to validate the biological activity of leptospiral proteins [17,18,19]. The saprophyte *Leptospira* has been employed as a model for genetic manipulation and gain-of-function analysis. Although it lacks most virulence factors compared to pathogenic species, it shares the same mechanisms of protein exportation [20]. Saprophytic leptospires also have a faster growth in vitro, with a generation time of ~4 h, reaching stationary phase in 2–3 days, while the generation time for pathogenic strains is approximately 20 h and the medium saturates in 4–7 days [21,22].

In this work, we used the *lic11711* gene, which is located close to other virulence related genes, i.e., those from the Lvr operon [23]. This operon codes for two hybrid signaling proteins: LvrA (LIC11709) and LvrB (LIC11708), which are only present in the pathogenic species of *Leptospira* [24]. The LIC11711 has its amino acid coding sequence conserved among other pathogenic species, with an identity above 96%, according to the BLASTp alignment [25]. The transcript level of this gene is higher in the virulent *L. interrogans* strain FIOCRUZ L1-130 compared with the culture-attenuated *L. interrogans* strain M20 [25]. The *lic11711* gene of *L. interrogans* serovar Copenhageni was cloned and the protein expressed in *Escherichia coli*. The recombinant protein was characterized in vitro as a surface protein and expressed during bacterial infection. The recombinant protein rLIC11711 was capable to interact with several ECM components, cellular receptors, fibrinolytic molecules, and components of the complement system, including Plg/Pla and Lam [25]. It seems that this protein has the potential to contribute to host-pathogen interactions in pathogenic *Leptospira* and therefore we decided to further investigate these functions in a bacterial cell system.

Here, we examined the gain-of-function approach by genetically fusing the *lic11711* gene with the *lipL32* promoter (P32) to overexpress the protein in *L. biflexa* serovar Patoc. The P32 is a strong promoter used to express proteins in heterologous systems [14,19]. In addition, LipL32 is a major lipoprotein of pathogenic *Leptospira*, expressed at high levels both during cultivation and during infection, with a putative role during mammalian infection [26].

The results strengthen the previous in vitro characterization of the purified recombinant LIC11711, indicating that this protein is surface-exposed in *L. interrogans*. The transformed *L. biflexa* acquired the capacity to interact with Lam and the Plg/Pla system, strongly suggesting that this protein participates in bacterial adhesion and promotes the proteolytic activity of the bacteria.

## 2. Results

### 2.1. P32LIC11711 Genetic Fusion and Plasmid Construction

**Figure 1:**
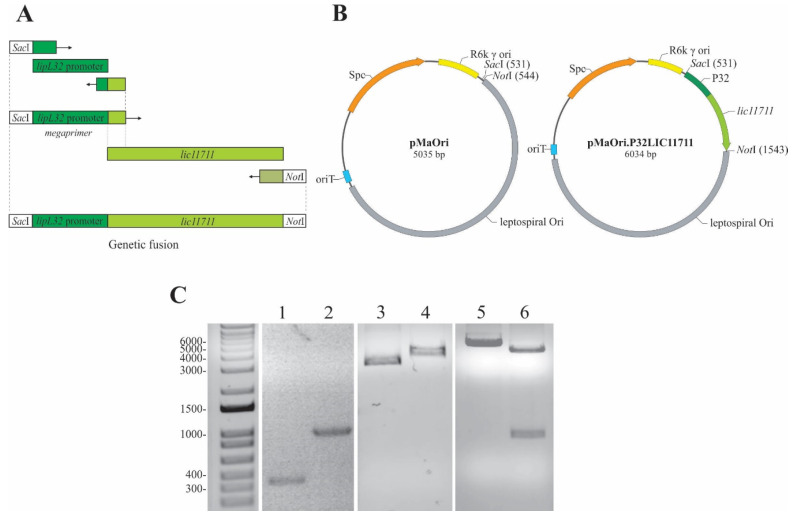
Scheme of genetic fusion and plasmid construction. (**A**) Representative scheme of the genetic fusion of P32 promoter with *lic11711* gene of *L. interrogans* by PCR. (**B**) Diagram of pMaOri and pMaOri.P32LIC11711 plasmids used to transform *L. biflexa*. (**C**) Analysis of the amplicons obtained in the construction steps of plasmid pMaOri.P32LIC11711 by 1% agarose gel electrophoresis. Lanes: 1—P32 promoter, 2—P32LIC11711 fusion, 3—pMaOri, 4—linearized pMaOri (digested with SacI), 5—pMaOri.P32LIC11711, 6- pMaOri.P32LIC11711 double-digested with SacI and NotI and insert release.

LIC11711 has its amino acid coding sequence conserved among other pathogenic species, with an identity above 96%, according to BLASTp alignment [25]. The combination of a strong constitutive gene promoter with a target gene that is poorly expressed is a strategy used to increase its heterologous expression in *Leptospira* spp. LipL32 is the most abundant protein of *L. interrogans*, with approximately 38,000 copies/cell [16] and its promoter is a great candidate for improving protein expression. Amplification of the P32 promoter from *L. interrogans* genomic DNA resulted in an approximately 350 bp PCR product (Figure 1A,C lane 1) and was used as a forward megaprimer for *lic11711* gene amplification, resulting in a genetic fusion of the target gene with the P32 promoter (P32LIC11711) (Figure 1A), with expected size of ~1000 bp (Figure 1B,C lane 2).

The pMaOri shuttle vector is 5035 bp long, which could be observed in the 1% agarose gel after its linearization with *Sac*I (Figure 1B,C, lane 4) and showing a gel migration of ~4000 bp when circular (Figure 1C, lane 3). The insertion of P32LIC11711 resulted in the pMaOri.P32LIC11711 vector 6034 bp long (Figure 1B,C, lane 5). A double digestion with *Sac*I and *Not*I of this recombinant plasmid resulted in the linearized pMaOri and P32LIC11711 fragments (Figure 1B,C, lane 6), confirming the insert ligation into pMaOri.

### 2.2. Heterologous Expression of LIC11711 under lipL32 Promoter in L. biflexa

After electroporation with the plasmids, colonies of recombinant *L. biflexa* were selected from Ellinghausen-McCullough-Johnson-Harris (EMJH) medium plates containing spectinomycin and cells were grown in liquid media containing the antibiotic for plasmid selection, once the LIC11711 expression system is episomal.

**Figure 2:**
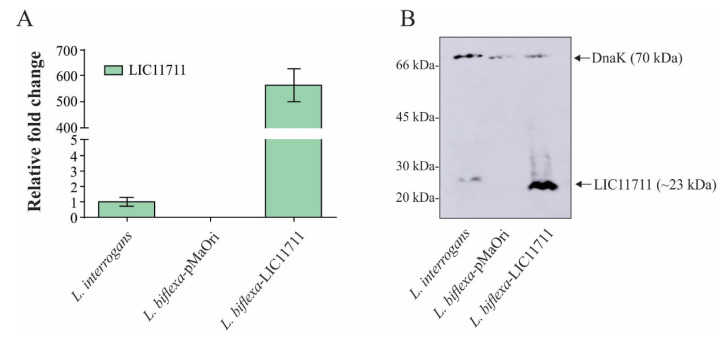
Heterologous expression of LIC11711 in *L. biflexa***.** (**A**) Total RNA *from L. interrogans*, *L. biflexa*-pMaOri and *L. biflexa*-LIC11711 were extracted and cDNA synthesized by reverse transcription. Transcription levels of *lic11711* gene were calculated by RT-qPCR and relative fold change of *L. biflexa*-LIC11711 was compared to pathogenic *L. interrogans* using 2^−ΔΔCt^ method. The bars represent the mean ± standard deviation (SD) of triplicates from two independent experiments. (**B**) Evaluation of LIC11711 expression by whole-cell lysate western blotting. LIC11711 was detected with anti-LIC11711 antiserum (1:1000) plus HRP-conjugated anti-mouse antibody (1:5000). DnaK was employed as experimental control and detected with anti-DnaK antiserum (1:3000) plus horseradish peroxidase (HRP)-conjugated anti-mouse antibody.

To confirm the overexpression of LIC11711 at the transcriptional level, mRNA levels were indirectly measured by quantitative reverse transcription PCR (RT-qPCR) (Figure 2A). Transcription of *lic11711* under P32 promoter (*L. biflexa*-LIC11711) was ~580x higher than with its native promoter (wild-type *L. interrogans* strain M20). The functionality of P32 in the surrogate *L. biflexa* is in accordance with previous works [14,19]. Motility or morphological differences between wild *L. biflexa*, *L. biflexa*-pMaOri and *L. biflexa*-LIC11711 were not observed.

The heterologous expression of LIC11711 by transformed *L. biflexa* was further evaluated by Western blotting. In Figure 2B, a ~23-kDa band referring to LIC11711 was detected in the recombinant *L. biflexa* at a higher intensity than in pathogenic *L. interrogans*, where no bands were observed in *L. biflexa*-pMaOri. The cytoplasmic DnaK (~70-kDa band) [27] was used as experimental control, in all leptospiral lysates. In the Western blotting experiment, polyclonal anti-DnaK antiserum was raised against recombinant DnaK protein from *L. interrogans*. DnaK from *L. biflexa* possesses 80% similarity and this could account for the slightly higher signal in DnaK band of *L. interrogans*, even though the cellular extracts were normalized before gel application. Band intensities between Dnak in *L. biflexa*-pMaOri and in *L. biflexa*-LIC11711 were similar, which led us to speculate, at least for DnaK chaperone, that there was no upregulation due to LIC11711 overexpression.

Taken together, these results confirm the overexpression of LIC11711 under the control of the P32 promoter in *L. biflexa*, which were utilized as a system for determination of cellular localization and protein function.

### 2.3. LIC11711 Localization in L. biflexa by ELISA

**Figure 3:**
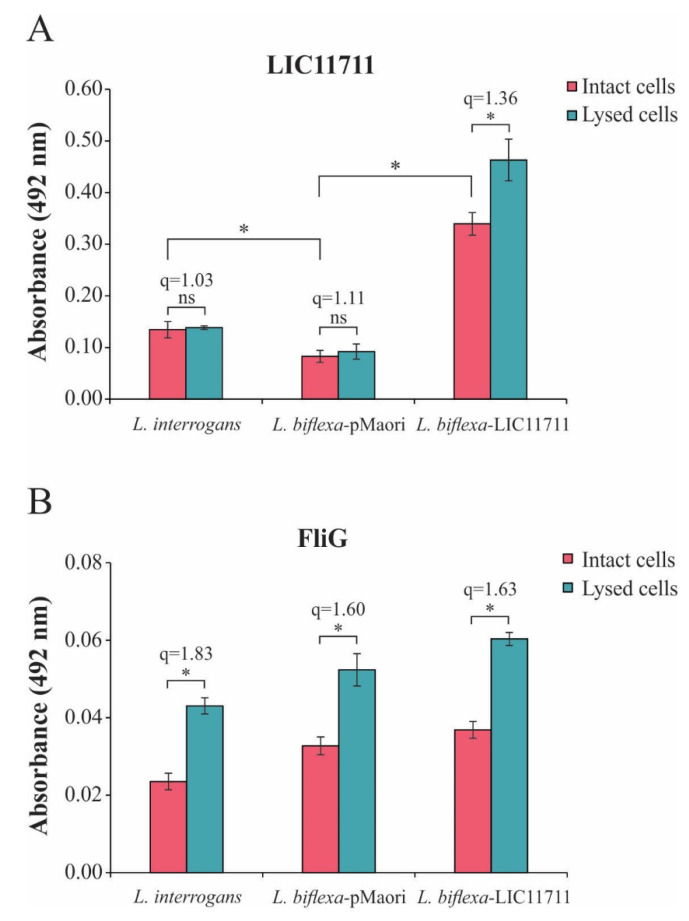
LIC11711 localization in recombinant *L. biflexa* by ELISA. Intact leptospires or total bacterial cell lysates were standardized at 0.1 optical density at 420 nm (OD_420nm_) and immobilized on the plate. (**A**) Protein detection was performed by incubating anti-LIC11711 antiserum (1:1000) followed by incubation with HRP-conjugated anti-mouse (1:5000) and detection with addition of o-phenylenediamine dihydrochloride (OPD) substrate. (**B**) Flagellar protein FliG was used as an experimental control. The results shown in the figure are from an independent experiment of two and the mean error bars were calculated based on an experimental quadruplicate. Statistical analysis was performed by Mann–Whitney *U* test (* *p* < 0.05) comparing the absorbances obtained between intact leptospira and total lysate within the same group (q values refer to the ratio). ns: not statistically significant.

The LIC11711 protein was characterized as a surface protein in *L. interrogans* by in vitro experiments [25]. To confirm the cellular localization of LIC11711, we performed an ELISA using intact leptospires or whole cell lysates. In Figure 3A, we observed an of 2.52-fold increase in absorbance in intact *L. biflexa*-LIC11711 compared to *L. interrogans*, reinforcing the expression and presence of LIC11711 on the leptospiral surface, while the detection signal was baseline for *L. biflexa*-pMaOri. There was no significant difference in LIC11711 detection signals in *L. interrogans* and *L. biflexa*-pMaOri when comparing intact or lysed cells, while there was a small increase in detection of LIC11711 in transformed *L. biflexa* (q = 1.36). Previous studies have shown that the machinery involved in *L. biflexa* protein exportation is highly conserved, being much the same as in *L. interrogans* [20], and is functional when other pathogen-specific genes are heterologously expressed [17,19]. We hypothesize that this slight increase after lysis is because the export machinery might be overwhelmed by the overexpression of LIC11711, and therefore, an amount of heterologous proteins remained in the cytosol.

The assay was also performed with FliG, a protein present in the rotor-mounted switch complex of leptospiral flagella (Figure 3B). Unlike LIC11711, there was an increase in detection of FliG when compared to lysed cells with intact cells (q ≥ 1.60), as expected from an inner membrane protein (Figure 3B). Taken together, these results suggest that LIC11711 is surface-exposed since absorbance remained similar even when cells were lysed, and cytoplasmic proteins were released.

### 2.4. L. biflexa-LIC11711 Adhesion to Lam

**Figure 4:**
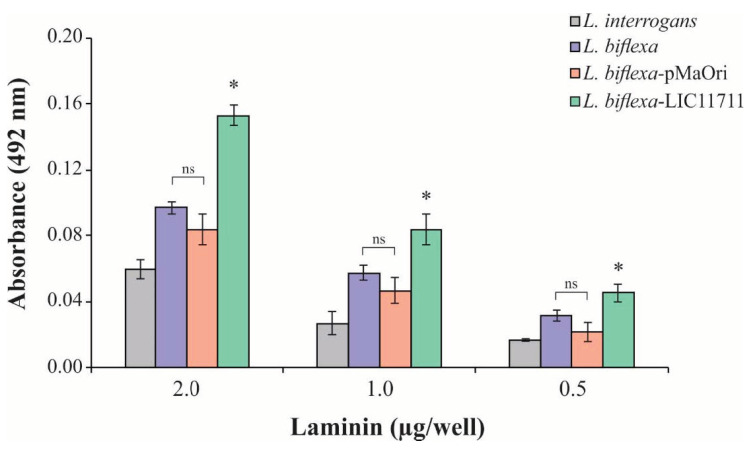
*L. biflexa*-LIC11711 interaction with Lam. *L. interrogans*, *L. biflexa*, *L. biflexa*-pMaOri, and *L. biflexa*-LIC11711 strains were standardized at 0.1 OD_420nm_ and immobilized on the plate and increasing concentrations of Lam were added. Interaction was measured by absorbance after incubation with anti-Lam (1:3000) and secondary antibody HRP-conjugated anti-rabbit (1:5000). The result represents an experiment out of two and bars represent mean ± SD, calculated from the experimental quadruplicate. For statistical analysis, the binding of *L. biflexa*-LIC11711 to Lam was compared with all other strains assayed, and wild-type *L. biflexa* was compared to *L. biflexa*-pMaOri by Mann–Whitney *U* test (* *p* < 0.05). ns: not statistically significant.

To determine the possible functional gains when expressing LIC11711, the interaction of *L. biflexa*-LIC11711 with Lam was examined, since the interaction with Lam has been described [25]. ELISA plates were coated with intact leptospiral cell suspension followed by addition of increasing concentrations of Lam. There was an increase in the binding of *L. biflexa*-LIC11711 to Lam compared to *L. biflexa* and transformed with empty pMaOri and *L. interrogans*, measured by absorbance intensity (Figure 4). The fact that there is lower binding of *L. interrogans* with Lam compared to *L. biflexa* is intriguing. One possibility is the fact that Lam is a sticky glycoprotein and, in this way, *L. biflexa* may have proteins that could adhere to this molecule. The interaction was dose-dependent, where absorbance increased with the amount of Lam available in the binding.

### 2.5. L. biflexa-LIC11711 Shows Enhanced Binding to Plg/Pla System

**Figure 5:**
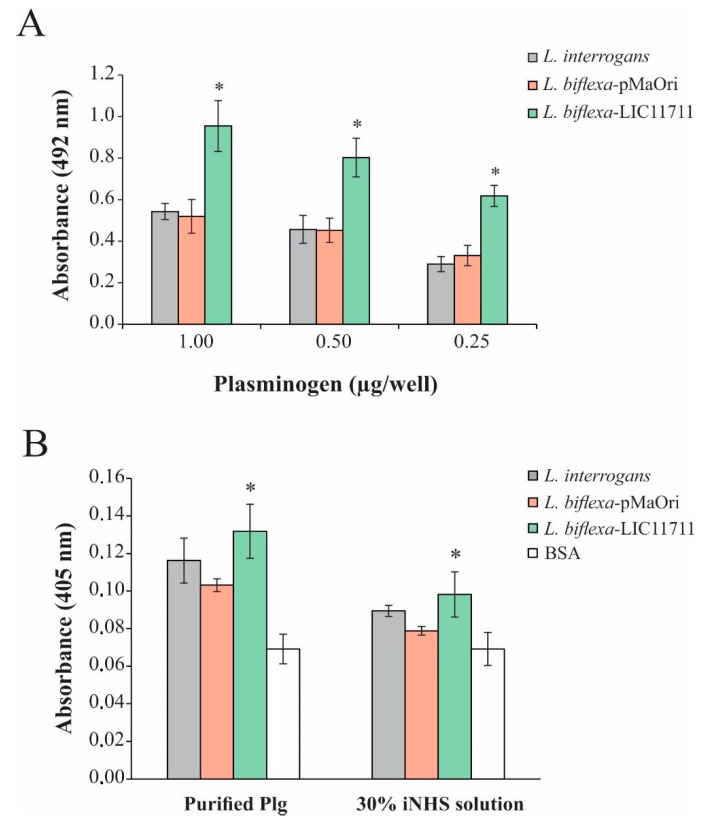
Plg binding and Pla activity by *L. biflexa*-LIC11711. (**A**) Binding of Plg to *L. interrogans*, *L. biflexa*-pMaOri and *L. biflexa*-LIC11711 strains was evaluated by dose-response assay using increasing concentrations of Plg. *L. interrogans*, *L. biflexa*-pMaOri, and *L. biflexa*-LIC11711 strains were standardized at 0.1 OD_420nm_, coated on the plate and increasing concentrations of Plg were added. Binding was measured in OD_492nm_ after incubation with anti-Plg (1:5000) and secondary antibody HRP-conjugated anti-mouse (1:5000). Bars represent mean ± SD of an independent assay out of two, calculated from the experimental quadruplicate. For statistical analysis, the binding of *L. biflexa*-LIC11711 and *L. biflexa*-pMaOri to Lam was compared by Mann-Whitney *U* test (* *p* < 0.05). (**B**) ELISA plates were coated with *L. interrogans*, *L. biflexa*-pMaOri or *L. biflexa*-LIC11711 and incubated with purified Plg or 30% iNHS solution, followed by addition of uPa and chromogenic substrate D-Val-Leu-Lys 4-nitroanilide dihydrochloride. BSA was used as experimental control and proteolytic activity was measured by absorbance at OD_405nm_. Bars represent mean ± SD calculated from the experimental quadruplicate from an independent experiment out of two. The obtained absorbance of *L. biflexa*-LIC11711 was compared to *L. biflexa*-pMaOri by Mann–Whitney *U* test (* *p* < 0.05).

In order to evaluate the binding of *L. biflexa* expressing LIC11711, ELISA plates were coated with intact leptospiral cell suspension followed by addition of increasing concentrations of Plg. The heterologous expression in *L. biflexa*-LIC11711 also enabled a strong and dose-dependent binding to Plg, a key component in the fibrinolytic system. The increase was almost twice all Plg concentrations compared to other strains tested, confirming the interaction of LIC11711 with this component (Figure 5A). Plg bound by *L. biflexa*-LIC11711 was able to be converted enzymatically to active Pla by exogenous activation with a urokinase plasminogen activator (uPa), and these Pla-coated leptospires were capable of degrading the chromogenic substrate D-val-leu-lys-p-nitroanilide dihydrochloride (Figure 5B). The proteolytic activity of Pla was proportionally the same when we used 30% NHS solution as a Plg source instead of commercial Plg, showing a higher proteolytic activity of Pla-coated *L. biflexa*-LIC11711. Interaction of Plg/Pla with *L. biflexa* [28] and with the recombinant protein LIC11711 [25] has already been reported. We show now that the highest capacity of *L. biflexa*-LIC11711 to bind Plg and Pla conversion is strong evidence that LIC11711 is capable of interacting with Plg/Pla within the *Leptospira* and thus potentially involved in virulence in pathogenic leptospiral strains.

### 2.6. Heterologous Expression of LIC11711 in L. biflexa Is Not Sufficient to Provide Serum Resistance

Recombinant LIC11711 was shown to interact with human complement C8 [25], which could account for the serum resistance displayed by pathogenic strains. Accordingly, antibody blockage of native LIC11711 in *L. interrogans* surface resulted in a low increase in serum susceptibility, suggesting the importance of this protein in binding some complement system components as an evasion mechanism [25].

**Figure 6:**
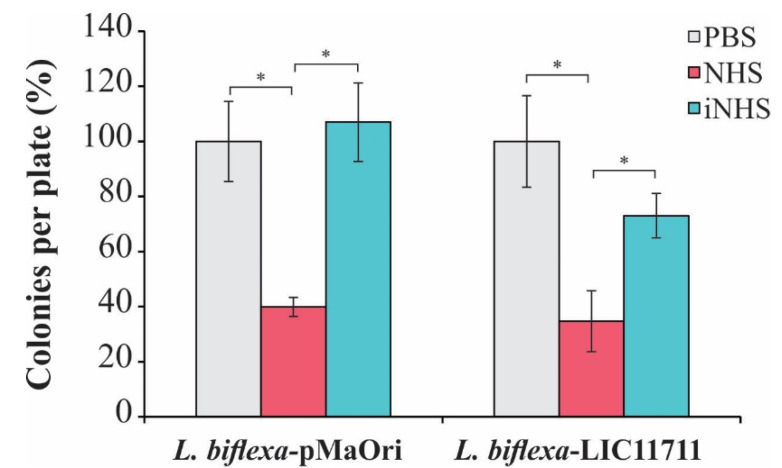
Effect of heterologous expression of LIC11711 on survival of *L. biflexa* after challenge with human serum. *L. interrogans*, *L. biflexa*-pMaOri, and *L. biflexa*-LIC11711 were standardized at 2 × 10^7^ cells/mL and incubated with 20% normal human serum (NHS), 20% inactivated normal human serum (iNHS), or phosphate-buffered saline (PBS) solutions. Cells were diluted to a final concentration of 10^3^ cells/mL and plated into EMJH agar medium. Serum resistance was verified by counting survival colonies and comparing between treatments (percentage relative to *L. interrogans*) by Mann-Whitney *U* test (* *p* < 0.05). Results represent an independent experiment of two and the mean and SD were calculated from a triplicate.

Accordingly, we evaluated whether the expression of LIC11711 could increase the survival of *L. biflexa* upon in vitro serum challenge. As shown in Figure 6, there was no statistical difference in the number of *L. biflexa*-LIC11711 colonies recovered compared to *L. biflexa*-pMaOri (control), showing that heterologous expression did not favor the survival of saprophytic leptospires against human serum. This result could be explained by the low affinity of recombinant LIC11711 to C8 and vitronectin and these weak interactions are not important with regard to virulent leptospiral resistance to host serum effects [25].

## 3. Discussion

Genetic manipulation is a strategy used to study the biology of pathogenic microorganisms and their virulence factors, which are important in host-pathogen interactions. The number of genetic tool available for *Leptospira* has increased over the years but is still limited when compared to other pathogenic bacteria [29].

The development of replicative plasmids for saprophytic *L. biflexa* [9], along with the further construction of the pMaOri vector for all leptospiral strains [10], has increased the possibility for the emergence of new strategies for genetic modifications in *Leptospira* spp. These genetic tools make it possible to study genes/proteins that might be relevant to bacterial pathogenicity and confirmation of phenotypes only supported by in vitro experiments.

The main barrier for studying the functional role of proteins of pathogenic leptospires relies on their functional redundancy, which does not always result in loss-of-function phenotypes after knockdown or knockout. In this sense, the heterologous expression of proteins in a genetically similar organism, such as *L. biflexa*, is an interesting approach to circumvent this problem, since the machinery involved in protein processing and exportation is similar compared to *L. interrogans* [20]. In addition, overexpression of heterologous proteins due to genetic combination with strong native promoters could render more evident phenotypes, which does not necessarily reflect the condition of natural infection but may be proportional to the number of copies of the native protein in the pathogenic leptospire.

The LigA and LigB proteins were expressed in the surrogate *L. biflexa* under regulation of the *flgB* promoter of *Borrelia burgdorferi*, and the recombinant leptospires showed increased binding to extracellular matrix components and MDCK monolayers [17]. Recombinant *L. biflexa* showed the ability to infect macrophages when heterologously expressing the MCE (mammalian cell entry) protein, showing the in vitro characterization of this protein [18]. The heterologous expression of LMB26 under the control of P32 increased the binding capacity of *L. biflexa* to fibronectin [19].

In this study, LIC11711 protein was overexpressed in *L. biflexa* under the regulation of the strong P32 promoter, previously shown to be functional in this surrogate [14,19]. As expected, this promoter increased target gene transcription by nearly 600-fold, when compared to *L. interrogans*, which in turn was reflected in higher protein levels. Cellular localization assays confirmed the surface-exposed nature of LIC11711.

In bacterial infection, an adhesion step is crucial for the success of the pathogen. Pathogenic *Leptospira* species possess a number of surface proteins that allow binding to components of the host’s extracellular matrix, including Lam [30,31,32], a multi-adhesive glycoprotein found in basal lamina, which is exposed in skin lesions [33]. Recombinant LIC11711 can bind to Lam in a dose-response manner [25] and therefore we determined this interaction through its heterologous expression in *L. biflexa*. A considerable increase in the binding capacity of *L. biflexa*-LIC11711 compared to *L. biflexa*-pMaOri and to pathogenic *L. interrogans* was observed. The latter was unexpected and we hypothesize that the higher affinity of LIC11711 for this host component, along with its higher copy number in the recombinant *L. biflexa*, could account for this effect. Indeed, quantitative RT-PCR and western blotting show that the levels of LIC11711 mRNA and proteins are higher in *L. biflexa*-LIC11711 than *L. interrogans*.

As *L. biflexa* expressing LIC11711 is grown in media containing spectinomycin, *L. biflexa* containing empty pMaOri was employed as negative control in the experiment once it grows in the same conditions. It is worth mentioning that no difference in binding to Lam was observed between *L. biflexa* transformed with the empty pMaOri vector and wild-type *L. biflexa*. Previous studies using the surrogate *L. biflexa* expressing virulent-related proteins focused the comparison of protein expression with the wild type *L. biflexa* [17,19]. In our study, we included pathogenic *L. interrogans* for comparative purposes. Although the information that there is no difference in the binding to Plg/Pla by *L. interrogans* and by *L. biflexa* is not novel (this was previously shown [28]), the case of Lam is particularly intriguing, and exhibited in this work for the first time. One possibility is the fact that Lam is a sticky glycoprotein that could “adhere” to *L. biflexa* proteins and this may account for the increased binding observed.

The fibrinolytic system is composed of several proteases and inhibitors that regulate the generation of Pla, a serine protease whose main function is fibrin degradation, thereby ensuring the maintenance of homeostasis [34]. Several microorganisms, including pathogenic *Leptospira* species, are known to be able to bind Plg and induce the secretion of activators by endothelial cells, thereby generating surface-bound PLA as a form of tissue penetration and immune system evasion [35]. Several leptospiral OMPs have been characterized as being capable of binding to Plg/Pla, including rLIC11711 [25,36,37,38,39,40].

*L. biflexa* expressing LIC11711 displayed increased binding to Plg in comparison to cells containing pMaOri alone, which in turn rendered higher Pla activity after uPa addition, when Plg either purified or from normal human serum (NHS) were employed as a zymogen source. These results strengthen the importance of this protein in Pla generation, which could enhance *L. interrogans* infection through tissue invasion and immune system evasion [35,41].

Currently, high-throughput strategies have been employed to elucidate how coding regions respond to environmental and/or host cues. RNAseq data from transcripts derived from virulent *L. interrogans* recovered from a dialysis membrane chamber (DMC) implanted in rat peritoneum have shown that LIC11711 mRNA is detected at similar levels as those from an in vitro cultivated virulent strain [42]. The presence of LIC11711 transcripts in DMC, a condition that mimics host infection more, indicated that the corresponding protein could contribute to leptospiral virulence.

Saprophytic leptospires do not have the ability to infect the target host and cannot cause leptospirosis. In contrast, pathogenic species have a number of surface proteins that are unique to these strains and can interact with the host; this difference is essential for successful infection [20,43]. Some characterized outer membrane leptospiral proteins show importance in recruiting host complement proteins and regulators [44,45] as a strategy to provide resistance against host serum. The heterologous expression of Lig proteins enhanced the survival of *L. biflexa* in human serum compared to wild type strain [46] and therefore we evaluated if *L. biflexa*-LIC11711 could present a similar phenotype gain.

Heterologous LIC11711 did not provide an increase in *L. biflexa* survival when in contact with normal human serum, as the number of recovered colonies after challenge were similar to *L. biflexa*-pMaOri. The low affinity of LIC11711 for complement system components and regulators [25] is most likely the reason for serum vulnerability of recombinant *L. biflexa*. As reported in the literature, other OMPs are potentially involved in the serum resistance of pathogenic leptospires by binding complement components and regulators [36,45,47].

In this study, we report heterologous expression of a pathogen-specific leptospiral protein in the surrogate *L. biflexa* and the characterization of its functionality in virulence mechanisms. This approach allowed the demonstration of LIC11711 as a potential virulence factor involved in adhesion and Plg/Pla acquisition, thereby increasing the proteolytic power of *L. interrogans*. This work reinforced the use of *L. biflexa* as a surrogate host to describe the role of crucial virulence factors of the etiological agent of leptospirosis.

## 4. Materials and Methods

### 4.1. Bacterial Strains and Culture Conditions

In this study, pathogenic culture-attenuated *L. interrogans* serovar Copenhageni strain M20 and the non-pathogenic *L. biflexa* serovar Patoc strain Patoc1 were used. Leptospires were cultured at 30 °C under agitation (80 rpm) and aerobic conditions in liquid EMJH medium [48] supplemented with Leptospira Enrichment EMJH (Difco, Franklin Lakes, NJ, USA). Spectinomycin (40 µg/mL) was added to culture when appropriate. *Escherichia coli* strain π1 [49] was used as plasmid cloning host and was grown in Luria-Bertani (LB) medium supplemented with thymidine (0.3 mM) and spectinomycin (40 µg/mL) at 37 °C.

### 4.2. Genetic Fusion and Plasmid Construction

The P32 was genetically fused to *lic11711* CDS by PCR. The first PCR was performed with the oligonucleotide P32 F and P32lic11711 R (Table 1) for P32 amplification. The product of this PCR was used as a megaprimer [50] in a second PCR for the genetic fusion of the amplified P32 with the *lic11711* gene using the lic11711 R oligonucleotide. Purified genomic DNA of *L. interrogans* serovar Copenhageni was used as template in all amplification steps. The resulting PCR product was digested with *Sac*I and *Not*I restriction enzymes at 37 °C for 2 h, purified with Illustra GFX PCR DNA and Gel Band Purification Kit (GE Healthcare, Chicago, IL, USA). The PCR product was inserted into the pMaOri vector [10], previously digested with the same restriction enzymes. The plasmid was a kind gift from Dr. Mathieu Picardeau (Biology of Spirochetes Unit, Institute Pasteur, Paris, France). *E. coli* strain π1 was transformed by heat shock with ligation reaction and plated onto LB medium containing thymidine (0.3 mM) and spectinomycin (40 µg/mL). Colonies were harvested and evaluated for the presence of recombinant plasmid by colony PCR, with pMaOri set of primers (Table 1). Positive colonies were grown for 16 h under shaking at 37 °C in 5 mL of LB medium, which was supplemented with thymidine (0.3 mM) and spectinomycin (40 µg/mL). Cells were harvested by centrifugation (3500× *g*). pMaOri.LIC11711 was purified using Illustra plasmidPrep Mini Spin Kit (GE Healthcare) and stored at −20 °C. All plasmids were fully sequenced before leptospiral transformation. Experiments were carried out only when no mutation was observed in the sequences.

### 4.3. L. biflexa Transformation

The transformation of saprophytic *L. biflexa* with pMaOri.LIC11711 or empty pMaOri was performed by electroporation as described elsewhere [22]. Briefly, *L. biflexa* was grown in EMJH medium at 30 °C until reaching exponential phase (0.3–0.5 at OD_420nm_). Cells were harvested by centrifugation (3500× *g*, room temperature) and washed in same volume with sterile water. After centrifugation, cells were resuspended in sterile water to a concentration of approximately 10^10^ cells/mL and 100 µL of bacterial suspensions were mixed with the plasmid. The mixture was added to a 0.2 mm cuvette (chilled at −20 °C) and electroporation was performed with the parameters 1.8 kV, 25 µF and 200 Ω. One milliliter of fresh liquid EMJH was added and cells were incubated at 30 °C with shaking. The transformed cells were plated on solid EMJH medium plus 40 µg/mL spectinomycin and incubated at 30 °C until colonies were visible.

Colonies were selected, grown in liquid medium, and PCR was then performed using pMaOri F and pMaOri R oligonucleotides (see Table 1) to confirm the presence of the plasmids. Recombinant *L. biflexa* was maintained by serial passage in liquid EMJH with spectinomycin.

### 4.4. RNA Extraction and Real-Time Reverse Transcriptase Quantitative PCR (RT-qPCR)

*L. interrogans* serovar Copenhageni, *L. biflexa* transformed with empty pMaOri (*L. biflexa*-pMaOri) and *L. biflexa* transformed with pMaOri.LIC11711 (*L. biflexa*-LIC11711) were harvested from EMJH liquid medium, and total RNA was extracted by Trizol reagent, according to the manufacturer’s instructions. Samples were treated with DNAse I (0.1 U/mL, Invitrogen, Carlsbad, CA, USA) for 1 h at room temperature to remove residual DNA. cDNAs were synthetized from total RNA by reverse transcription using the SuperScript III Reverse Transcriptase kit (Invitrogen, Carlsbad, CA, USA).

RT-qPCR was performed with SYBR Green PCR Master Mix (Applied Biosystems, Foster City, CA, USA) and oligonucleotides qPCRlic111711 F and qPCRlic11711 R (Table 1) in a 20-µL reaction volume, using the CFX96 Real-Time System (Bio-Rad, Hercules, CA, USA) with the following parameters: 10 min at 95 °C; 40 cycles of 15 s at 95 °C and 1 min at 58 °C. The relative expression of *lic11711* was measured using the 2^-ΔΔCt^ method [51] and normalized with *L. interrogans* 16S rRNA expression.

### 4.5. Polyclonal Antiserum Production

The heterologous expression and purification of recombinant LIC11711, DnaK, and FliG (LIC10023) and homologous polyclonal antiserum production was performed as previously described [14,25]. Briefly, BALB/c mice (4–6 weeks old) were immunized subcutaneously with 10 µg of each recombinant protein in 10% (*v*/*v*) of Alhydrogel adjuvant [2% Al(OH)_3_; Brenntag Biosector]. Two subsequent booster immunizations were performed at 2-week intervals. Mice were bled from the retro-orbital plexus 14 days after each immunization. The titers of pooled anti-recombinant protein sera were determined by ELISA. The Ethics Committee for Animal Research of Butantan Institute, Brazil, approved all animal studies, registered under protocol No.1389020518.

### 4.6. Validation of LIC11711 Expression in L. biflexa by Western Blotting

Late-log phase cultures of *L. interrogans* serovar Copenhageni, *L. biflexa*-pMaOri, and *L. biflexa*-LIC11711 were recovered from EMJH by centrifugation (3500× *g*), washed twice with phosphate-buffered saline (PBS), and resuspended in the same buffer. Cell samples were standardized to the same A_420nm_, mixed with denaturation buffer (50% glycerol, 10% SDS, 3% β-mercaptoethanol, 0.5% bromophenol blue), and heated for 10 min at 96 °C. The whole-cell lysates were separated by 15% SDS-polyacrylamide gel electrophoresis (SDS-PAGE) and proteins transferred to a nitrocellulose membrane. The membrane was blocked with PBS plus 0.05% A Tween-20 and 5% nonfat dry milk solution (PBS-T/milk) was set at 4 °C overnight. The membrane was incubated with polyclonal anti-serum raised against recombinant LIC11711 (1:1000), followed by incubation with horseradish peroxidase (HRP)-conjugated anti-mouse IgG (anti-mouse) (1:5000). For the experimental control, we determined cytoplasmic protein DnaK [27], incubating membrane with polyclonal anti-DnaK (1:3000) followed by anti-mouse incubation. Detection was performed using Super Signal West Dura Extended Duration Substrate (Thermo Fisher Scientific, Waltham, MA, USA).

### 4.7. LIC11711 Localization in L. biflexa by ELISA Using Intact and Lysed Cells

ELISA plate wells were coated with 100 µL/well of intact or lysed *L. interrogans*, *L. biflexa*-pMaOri and transformed *L. biflexa*-LIC11711, standardized at A_420nm_ 0.1 and incubated at 30 °C overnight. Unbound leptospires were removed by washing three times with PBS 1x and plates were blocked with 200 µL of PBS containing 1% bovine serum albumin (BSA) (PBS/BSA) at 30 °C for 2 h. Cells were incubated for 1 h at 30 °C with 100 µL of polyclonal anti-rLIC11711 antiserum (1:1000) in PBS/BSA solution followed by incubation with secondary anti-mouse antibody (1:5000 for 1 h at 30 °C). Colorimetric reaction was performed by adding 1 mg/mL ο-phenylenediamine dihydrochloride (OPD, Sigma-Aldrich, St. Louis, MO, USA) in citrate a phosphate buffer (pH 5.0) plus 1 μL/mL H_2_O_2_. The reaction was carried out for 5 min and stopped by the addition of 50 μL 2 M H_2_SO_4_. We also detected the flagellar motor switch protein FliG, located in the inner membrane facing the cytoplasm [52].

For experimental control, the anti-rLIC11711 or anti-rFliG antiserum was omitted in each treatment. The mean values and standard deviation are representative of an experiment performed with independent triplicates, where the absorbance values in the tests are subtracted from the values when anti-rLIC11711 or anti-rFliG was omitted. The ratio (q) between the absorbance of intact cell treatment and lysed cell treatment was calculated to illustrate the relative increase in detection of LIC11711 or FliG.

### 4.8. Binding Assays

ELISA plate wells were coated with 100 µL of intact leptospiral cell suspension, standardized at 0.1 (A_420nm_), and incubated at 30 °C overnight. The plate wells were washed three times with PBS and blocked with 200 µL of PBS containing 1% bovine serum albumin (BSA; *m*/*v*) (PBS/BSA) at 30 °C for 2 h. Increasing concentrations of the purified components were then added individually to interact with coated leptospires at 30 °C for 2 h. The plates were washed three times with PBS to remove unbounded components, and 100 µL of anti-component antibody were added per well (*in house* mouse anti-Plg: 1:5000; rabbit anti-Lam: 1:3000; Sigma-Aldrich, San Luis, MO, USA) for 1 h at 30 °C. After washing, secondary antibodies, HRP-conjugated anti-mouse or rabbit, were added (1:5000). The colorimetric reaction and absorbance readings were performed as described above.

For statistical analysis, the mean values of each component interaction with *L. biflexa*-LIC11711 were compared with the mean of the binding to *L. biflexa*-pMaOri type, using the Mann–Whitney *U* test and the *p*-value below 0.05 was considered statistically significant.

### 4.9. Pla Generation by L. biflexa-LIC11711-Bound Plg

ELISA plate wells were coated with leptospires, standardized as described above and incubation occurred at 30 °C overnight. The plate was washed 3 times with PBS and blocked with PBS/BSA solution at 30 °C for 2 h. Next, 1 μg of Plg or PBS containing 30% (*v*/*v*) inactivated human serum (iNHS; heating at 56 °C for 20 min) was added and the plate incubated for 2 h at 30 °C. In sequence, 4 ng of uPa and the chromogenic plasmin substrate D-Val-Leu-Lys 4-nitroanilide dihydrochloride (0.4 mM; Sigma) were added to each well. The plates were incubated at 37 °C and substrate degradation was determined at different times measuring absorbance at 405 nm.

For experimental control, the absorbance values of each treatment were compared with the absorbance values of the control treatments, where Plg, uPa or substrate was omitted. For statistical analysis the absorbance values of *L. biflexa*-LIC11711 were compared with *L. biflexa*-pMaOri using the Mann–Whitney *U* test, and a *p*-value below 0.05 was considered statistically significant.

### 4.10. Serum Resistance Assay

*L. biflexa*-pMaOri and *L. biflexa*-LIC11711 were recovered from EMJH medium by centrifugation (3500× *g* 15 min) and standardized to 2 × 10^7^ cells/mL. Samples of 100 µL were incubated with 100 µL of 20% NHS, 20% iNHS solution or PBS for 2 h at 30 °C. After incubation, cells were diluted and 100 µL of a 10^3^ cells/mL suspension were plated on EMJH solid medium. Plates were monitored daily until colonies appeared (around 10 days) and the effect of LIC11711 expression on *L. biflexa* survival was determined by comparing with *L. biflexa*-pMaOri incubated with NHS by colony counting. For statistical analysis, the number of colonies grown was compared between treatments using the Mann–Whitney *U* test, and a *p*-value below 0.05 was considered statistically significant.

## Figures and Tables

**Table 1:** List of oligonucleotides used in this study. The underline sequences refer to the restriction sequences.

Oligonucleotide	Sequence (5′→3′)	Restriction Site
P32 F	GAGCTCGAACAAGAAAGAGTCAGAG	*Sac*I
P32-lic11711 R	GGATTTTGATTTTATAGCCGACATAGACTCTCCTTAGTTAG	
lic11711 R	GCGGCCGCTTATTTTCTGCGAATCACTTC	*Not*I
pMaOri F	AGTGACACAGGAACACTTAACG	
pMaOri R	TATATTCTGTCCACATTTGTGG	
qPCRlic11711 F	AAAGGGGGAGACGTTTTGAT	
qPCRlic11711 R	CGTTTCAAATGCTCCCGTAT	
